# Corneal higher-order aberrations in corneal endothelial decompensation secondary to obstetric forceps injury

**DOI:** 10.1038/s41598-023-32683-5

**Published:** 2023-04-03

**Authors:** Hirotsugu Kasamatsu, Yukari Yagi-Yaguchi, Takefumi Yamaguchi, Sota Nishisako, Toshinori Murata, Jun Shimazaki

**Affiliations:** 1 Department of Ophthalmology, Tokyo Dental College Ichikawa General Hospital, 5-11-13, Sugano, Ichikawa, Chiba 272-8513 Japan; 2 Department of Ophthalmology, Shinshu University School of Medicine, Nagano, Japan; 3 Cornea Center and Eye Bank, Tokyo Dental College, Ichikawa General Hospital, Chiba, Japan

**Keywords:** Medical research, Eye diseases

## Abstract

Forceps corneal injuries during infant delivery cause Descemet membrane (DM) breaks, that cause corneal astigmatism and corneal endothelial decompensation. The aim of this study is to characterise corneal higher-order aberrations (HOAs) and corneal topographic patterns in corneal endothelial decompensation due to obstetric forceps injury. This retrospective study included 23 eyes of 21 patients (54.0 ± 9.0 years old) with forceps corneal injury, and 18 healthy controls. HOAs and coma aberrations were significantly larger in forceps injury (1.05 [0.76–1.98] μm, and 0.83 [0.58–1.69], respectively) than in healthy controls (0.10 [0.08–0.11], and 0.06 [0.05–0.07], respectively, both *P* < 0.0001). Patient visual acuity was positively correlated with coma aberration (*r*_*s*_ = 0.482, *P* = 0.023). The most common topographic patterns were those of protrusion and regular astigmatism (both, six eyes, 26.1%), followed by asymmetric (five eyes, 21.7%), and flattening (four eyes, 17.4%). These results indicate that increased corneal HOAs are associated with decreased visual acuity in corneal endothelial decompensation with DM breaks and corneal topography exhibits various patterns in forceps injury.

## Introduction

Forceps corneal injuries during infant delivery were first reported by Noyes et al.^1^ in 1895. Forceps corneal injury is characterised by Descemet membrane (DM) breaks, occurring vertically, and obliquely oriented striae on the inner corneal surface. After delivery, corneal oedema is obvious, however, spontaneously resolves within a few weeks or months, leaving visible edges of the break and a clear cornea. These breaks have been associated with various degrees of asymmetric astigmatism, and deep amblyopia^2^. Slowly progressive endothelial cell loss and secondary corneal decompensation ensue, causing corneal oedema and bullous keratopathy in the fourth or fifth decade of life^3^. Forceps corneal injury is responsible for 1.6% of all bullous keratopathy cases^4^, and 2.0% of the indications for Descemet stripping automated endothelial keratoplasty (DSAEK) in Japan^5^. Although several case studies have reported successful DSAEK for the treatment of bullous keratopathy due to forceps corneal injury^6–8^, careful planning is needed because amblyopia, high levels of astigmatism, an irregular posterior corneal surface, and severe corneal oedema may be present. Furthermore, there are intraindividual differences in these properties. However, the structural/optical features of the cornea have not been well investigated.

Recent technological advances have allowed the acquisition of images and precise information on the cornea and the anterior portion of the eye^9–12^. Anterior segment optical coherence tomography (AS-OCT) is used to obtain precise data even in opaque corneas^12,13^. Using AS-OCT, we recently reported that presence of higher-order aberrations (HOAs) were significantly associated with reduction in visual acuity in eyes with severe corneal opacity, such as herpes simplex keratitis^14^, corneal stromal dystrophies^15^, and Stevens-Johnson syndrome^16^. We observed that patients with bullous keratopathy due to forceps corneal injury exhibited several typical corneal topographic patterns, similar to those seen in other corneal diseases^14–16^. Thus, we hypothesized that forceps corneal injury causes increased HOAs, leading to decreased visual acuity, and exhibits several topographic patterns; when evaluating the influence of corneal optical property on visual function and determining corneal transplantation in eyes with forceps corneal injury, HOAs and topographic patterns can be an objective biomarker. In the current study, we first calculated corneal HOAs in eyes with bullous keratopathy due to forceps corneal injury and compared them with HOAs present in healthy eyes. Second, we characterised the topographic patterns of bullous keratopathy due to forceps corneal injury. Third, we evaluated the correlation between visual acuity and HOAs in these eyes, with the aim of identifying the effects of HOAs on visual acuity. Fourth, we analysed HOAs before and after DSAEK for the treatment of forceps corneal injury.

## Methods

### Patients

This retrospective consecutive case series study was performed in accordance with the principles of the Declaration of Helsinki, and the study protocol was approved by the Institutional Ethics Review Board of Tokyo Dental College Ichikawa General Hospital (I-15-51). Our institutional review board waived the requirement for informed consent, and the patient data were anonymised before access and/or analysis. This study included 23 eyes of 21 consecutive patients (54.0 ± 9.0 [mean ± standard deviation: SD] years) who had been diagnosed with corneal endothelial decompensation due to forceps corneal injury at Ichikawa General Hospital from October 2009 to November 2018, and 18 eyes of age matched healthy control participants (Table 1). We excluded patients with corneal scarring caused by other conditions, such as trauma or infectious keratitis, and history of ocular surface surgeries that could induce corneal astigmatism, such as for pterygium, glaucoma surgery, and corneal transplantation. The mean age of the participants in the healthy control group was 55.1 ± 18.5 years.

**Table 1:** Patient demographics.

	Forceps corneal injuries	Healthy controls	*P* value*
No. of eyes	23	18	
Age, mean (SD), years	54.0 ± 9.0	51.1 ± 18.0	0.12^α^
Interval between on set age and measurement, m	4.9 ± 6.5		
Sex, Female, *n* (%)	13 (61.9)	13 (72.2)	0.63^α^
BSCVA, Median (IQR), logMAR	1.70 (0.76, 2.00)	0 (-0.06, 0)	< 0.01^β^
Average K value	43.7 ± 4.7	43.9 ± 1.5	0.56^β^
Cylinder (D)	3.9 ± 2.1	1.0 ± 0.7	< 0.01^β^
Ks (D)	45.6 ± 5.0	44.4 ± 1.5	0.28^β^
Kf (D)	41.9 ± 4.6	43.4 ± 1.5	0.55^β^
Amblyopia (%)	20/21 (95.7)		
CCT (μm)	819.7 ± 131.3	533.3 ± 34.7	< 0.01^β^
Opacity grading^a^	1.7 ± 1.0		
Grade 0,* n* (%)	3 (13.1)	18 (100)	
Grade 1, *n* (%)	6 (26.1)	0 (0)	
Grade 2, *n* (%)	9 (39.1)	0 (0)	
Grade 3, *n* (%)	5 (21.7)	0 (0)	
Bullous stage^b^	2.5 ± 1.1	0	
Stage 1, *n* (%)	3 (13.1)	0 (0)	
Stage 2, *n* (%)	10 (43.5)	0 (0)	
Stage 3,* n* (%)	5 (21.7)	0 (0)	
Stage 4, *n* (%)	5 (21.7)	0 (0)	
Astigmatism matched with DM rupture (%)	14 (60.9)	NA	

BSCVA, best-spectacle corrected visual acuity; CCT, central corneal thickness; DM, Descemet’s membrane; IQR, interquartile range; logMAR, logarithm of minimal angle resolution.

^a^Corneal opacity grade; 0, clear or trace haze; grade 1, mild opacity; grade 2, moderately dense opacity partially obscuring the details of the iris; and grade 3, severely dense opacity obscuring the details of the intraocular structures.

^b^The severity of bullous keratopathy was defined as follows: stage 1, slight stromal edema or increased corneal thickness due to reduction in corneal endothelial cells; stage 2, stromal edema with DM folds; stage 3, stromal edema and DM folds with apparent epithelial bullae; stage 4, stromal scarring and corneal neovascularization.

^α^*P* values: Pearson's chi-square test, ^β^*P* values: Mann–Whitney test.

### Data analysis

Routine testing of all patients included slit-lamp microscopy, fundus, and best-spectacle corrected visual acuity (BSCVA) examinations at the time of diagnosis. Visual acuity was measured using the standard Landolt optotype. The measured decimal acuity was converted to logarithm of the minimum angle of resolution (logMAR) units using a Visual Acuity Conversion Chart. We defined amblyopia as reduced visual acuity with BSCVA below 20/20 without pathological causes such as cataracts and disorders in the macula and optic nerve^17^. A blinded observer (HK) graded the extent of corneal opacity and severity of bullous keratopathy based on the slit-lamp examination results according to a previously described system as follows^18^: corneal opacity grade; 0, clear or trace haze; grade 1, mild opacity; grade 2, moderately dense opacity partially obscuring the details of the iris; and grade 3, severely dense opacity obscuring the details of the intraocular structures. The severity of bullous keratopathy was defined as follows: stage 1, slight stromal oedema or increased corneal thickness due to reduction in corneal endothelial cells; stage 2, stromal oedema with DM folds; stage 3, stromal oedema and DM folds with apparent epithelial bullae; stage 4, stromal scarring and corneal neovascularisation.

### Anterior segment optical coherence tomography

AS-OCT was used to evaluate the corneal structure in eyes with forceps corneal injury as reported previously^14–16^. In brief, the CASIA system (SS-1000, Tomey, Nagoya, Japan) corrected distortions in the AS-OCT images based on the refractive index of the anterior surface. Two corneal specialists (HK and TY) carefully checked all AS-OCT images to ensure that the surface digitalisation recognised by the automated inbuilt software was correct. The anterior and posterior corneal surfaces were reconstructed as a three-dimensional model from the corneal height data. The anterior, posterior, and total corneal aberrations at the diameters of 4 mm and 6 mm were calculated separately with the installed ray tracing software, version 5.1. The refractive indices of the cornea and aqueous humor were set to 1.376 and 1.336, respectively. The wavefront aberration was expanded with normalized Zernike polynomials up to the 8th order. HOA was defined as the root mean square (RMS) of the 3rd- to 8th-order Zernike coefficients as follows^18^

$$\begin{aligned} HOA & = \sqrt {\sum\limits_{j = 3}^{20} {\left( {Z_{j} } \right)^{2} } } \;\;Z_{j} = Z_{n}^{m} \ldots j = \frac{n(n + 2)m}{2} \cdot n = roundup\left[ {\frac{{ - 3 + \sqrt {9 + 8j} }}{2}} \right] \\ m & = 2j - n(n + 2) \\ \end{aligned}$$

Spherical aberration (SA) was defined as the RMS of Z_4_^0^ (spherical aberration) and Z_6_^0^ (secondary spherical aberration). Coma aberration was defined as the RMS of Z_3_^–1^ and Z_3_^1^.

$$SA = \sqrt {Z_{4}^{{0^{2} }} + Z_{6}^{{0^{2} }} } \;\;\;Coma = \sqrt {Z_{3}^{{ - 1^{2} }} + Z_{3}^{{1^{2} }} }$$

### Classification of corneal shapes into five topographic patterns

Topographic patterns were categorised into five types, i.e., regular astigmatism, asymmetric, protrusion, flattening, and posterior irregular with minimal anterior irregularity, as previously reported^14–16^. Briefly, the AS-OCT images of corneas with forceps corneal injury showed a high degree of irregular astigmatism on the anterior and posterior surfaces. The regular astigmatism pattern was defined as regular astigmatism with an astigmatism power of ≥ 3 diopters. The asymmetric pattern was defined as a mirror-image with blue and red asymmetric color on the topographic map of the anterior surface, half with a keratometric value of ≥ 45 diopters and the other half with a keratometric value of ≤ 40 diopters. The protrusion pattern was defined as a centrally steep zone with a keratometric value of ≥ 50 diopters on the anterior surface. The flattening pattern was defined as a flattened cornea with a keratometric value of ≤ 35 diopters on the anterior surface. The posterior irregular pattern with minimal anterior surface changes was considered when there were minimal changes in the anterior surface.

### Surgical procedures

Among 23 eyes, 11 patients (11 eyes) underwent DSAEK. DSAEK was performed using the double-glide technique. Briefly, after sub-Tenon anesthesia with an injection of 2% lidocaine, a 4.5-mm temporal corneoscleral incision was made. Descemet stripping was performed with a reverse-bent Sinsky hook (Asico, Westmont, IL, USA). The recipient’s endothelium and Descemet’s membrane were carefully removed using forceps. Pre-cut donor grafts were trephined and the endothelial surface of the donor lenticle was coated with a small amount of viscoelastic material. Donor tissue was gently inserted into the anterior chamber using a Busin glide (Asico) and Shimazaki forceps (Inami, Tokyo, Japan). Air was carefully injected into the anterior chamber to unfold the graft. At 10 min after air injection, half of the air was replaced by balanced salt solution (Alcon, Fort Worth, TX, USA). At the end of the surgery, 2 mg subconjunctival betamethasone was administered. Corneal HOAs were evaluated 3 months after DSAEK.

### Statistical analysis

The data were analysed using the Prism software (ver. 6.04 for Windows; GraphPad Software, Inc., San Diego, CA, USA). The D’Agostino and Pearson omnibus normality test was used to assess whether the data showed a normal distribution. Pearson's chi-square and Mann–Whitney tests were used to compare the differences between patients with forceps corneal injury and healthy controls. Spearman’s correlations were performed to determine the association between logMAR and each parameter including coma aberrations and HOAs within 4 mm and 6 mm diameters, opacity grade, bullous stage, and CCT. To comparison between before and after DSAEK for corneal higher-order aberrations, Wilcoxon signed-rank tests were used. Power analysis was performed using G power 3.1 to determine the number of patients in the control groups. Our pilot data (forceps Injury; n = 10 vs. healthy controls; n = 10) showed large effect sizes (r > 0.84, Cohen’s d > 0.94) in all test sections. In the Mann–Whitney U test, a sample size of approximately 40 observations was sufficient to achieve 80% power to detect an effect size of Cohen’s d of 0.94 at an alpha level of 0.05. All tests were 2-tailed and a *P* value less than 0.05 was considered to indicate statistical significance.

## Results

### Patient demographics

**Figure 1:**
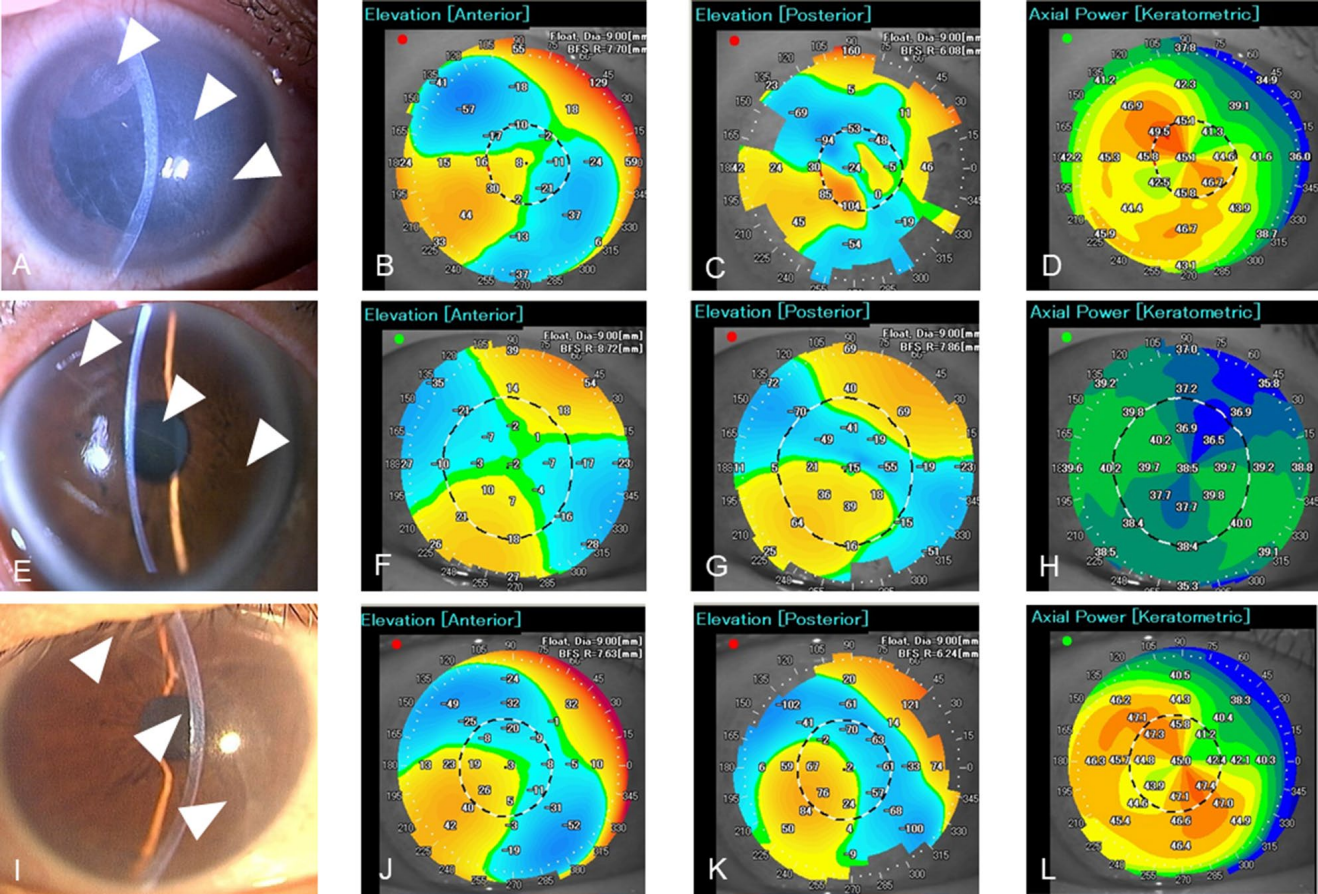
Direction of Descemet break and astigmatism axis. Case1 (**A**–**D**) is a 51-year-old woman, a Descemet membrane (DM) break appears obliquely present (**A**: arrow heads). Case2 (**E**–**H**) is a 69-year-old man, a Descemet membrane (DM) break appears horizontal present (**E**: arrow heads). Case 3 (**I**–**L**) is a 67-year-old woman, a Descemet membrane (DM) break appears vertical present (I: arrow heads). The topographic elevation map shows a blue area on the anterior (**B**, **F**, **J**) and posterior corneal surfaces (**C**, **G**, **K**) in the area with the DM break (**A**, **E**, **I**). Those blue areas are associated with the corneal astigmatism axis (**D**, **H**, **L**). In the current study, the direction of the DM breaks was identical to that of the corneal astigmatism axis in 14 of 23 eyes (60.9%).

Patient demographics are shown in Table 1. Of 23 eyes with forceps corneal injuries, 22 (95.7%) patients had amblyopia. The direction of DM break on slit photographs was horizontal (1–30°, 151–180°) in six eyes (26.1%), oblique (31–60°, 121–150°) in 12 eyes (52.2%), and vertical (61–120°) in five eyes (21.7%). The direction of DM breaks was identical to that of the corneal astigmatism axis of the steep meridian in 14 of 23 eyes (Fig. 1, 60.9%), whereas the direction of DM break was not associated with that of the corneal astigmatism axis of the steep meridian in the nine other eyes because of severe corneal deformations, such as asymmetric changes or protrusion.

### Higher-order aberrations in forceps corneal injuries

Compared with the healthy control eyes, all HOA parameters, including HOAs, SA, and coma aberrations (total/anterior/posterior, 4 mm/6 mm) were significantly higher in eyes with forceps corneal injury (Table 2, all *P* < 0.001). Total HOAs were larger than those of the anterior surface in forceps corneal injury.

**Table 2:** Corneal higher-order aberrations in bullous keratopathy due to forceps corneal injuries.

	Forceps corneal injuries (23 eyes)	Healthy controls (18 eyes)	*P* value*
HOAs 4 mm			
Total	1.05 (0.76, 1.98)	0.10 (0.08, 0.11)	< 0.001^α^
Anterior	0.92 (0.81, 1.69)	0.10 (0.09, 0.11)	< 0.001^α^
Posterior	0.50 (0.45, 0.77)	0.02 (0.02, 0.02)	< 0.001^α^
HOAs (6 mm)			
Total	1.79 (1.48, 2.83)	0.19 (0.16, 0.21)	< 0.001^α^
Anterior	2.03 (1.52, 2.82)	0.19 (0.17, 0.22)	< 0.001^α^
Posterior	0.87 (0.73, 1.06)	0.06 (0.05, 0.07)	< 0.001^α^
SA (4 mm)			
Total	0.72 (0.43, 1.03)	0.07 (0.05, 0.08)	< 0.001^α^
Anterior	0.61 (0.40, 0.92)	0.08 (0.06, 0.09)	< 0.001^α^
Posterior	0.34 (0.20, 0.45)	0.02 (0.01, 0.02)	< 0.001^α^
SA (6 mm)			
Total	1.00 (0.75, 1.51)	0.13 (0.11, 0.15)	< 0.001^α^
Anterior	0.99 (0.77, 1.43)	0.14 (0.12, 0.16)	< 0.001^α^
Posterior	0.42 (0.35, 0.57)	0.05 (0.05, 0.05)	< 0.001^α^
Coma (4 mm)			
Total	0.83 (0.58, 1.69)	0.06 (0.05, 0.07)	< 0.001^α^
Anterior	0.72 (0.53, 1.48)	0.06 (0.05, 0.07)	< 0.001^α^
Posterior	0.41 (0.30, 0.62)	0.01 (0.01, 0.01)	< 0.001^α^
Coma (6 mm)			
Total	1.35 (1.17, 2.57)	0.12 (0.10, 0.16)	< 0.001^α^
Anterior	1.72 (1.31, 2.62)	0.13 (0.11, 0.18)	< 0.001^α^
Posterior	0.72(0.55, 0.96)	0.02(0.02, 0.03)	< 0.001^α^

Data is shown by median (IQR) μm.

HOAs, higher-order aberrations; IQR, interquartile range; SA, spherical aberration.

^α^*P* values: Mann–Whitney test.

### Correlations among visual acuities, coma aberration, and opacity grade

LogMAR visual acuity was significantly positively correlated with coma aberrations in the 4 mm zone of the anterior surface, and 6 mm zones of the total cornea and anterior surface (Table 3, r_s_ = 0.454, *P* = 0.03; *r*_*s*_ = 0.482, *P* = 0.02; *r*_*s*_ = 0.504, *P* = 0.02, respectively). LogMAR visual acuity was positively correlated with the opacity grade and severity of the bullous stage (*r*_*s*_ = 0.700, *P* = 0.0003; *r*_*s*_ = 0.675, *P* = 0.0006, respectively), not with the CCT (r_s_ = 0.175, *P* = 0.435).

**Table 3:** Correlation between visual acuity, coma aberration, HOAs, opacity grade, bullous stage, and CCT.

	4 mm zone	6 mm zone
Coma aberration		
Total cornea	0.28 (0.21)	**0.482 (0.02)**
Anterior surface	**0.454 (0.03)**	**0.504 (0.02)**
Posterior surface	− 0.166 (0.46)	− 0.018 (0.94)
HOAs		
Total cornea	0.321 (0.14)	0.383 (0.071)
Anterior surface	0.374 (0.08)	**0.471 (0.02)**
Posterior surface	− 0.014 (0.95)	0.056 (0.80)
Opacity grade^*a*^	**0.700 (< 0.001)**	
Bullous stage^b^	**0.675 (< 0.001)**	
CCT	0.175 (0.43)	

Data are expressed as Spearman's correlation coefficient *r*_*s*_ (*P*).

Bold numbers indicate *P* < 0.05.

CCT, central corneal thickness; DM, Descemet’s membrane; HOAs, higher order aberrations; IQR, interquartile range; SA, spherical aberration.

^*a*^corneal opacity grade; 0, clear or trace haze; grade 1, mild opacity; grade 2, moderately dense opacity partially obscuring the details of the iris; and grade 3, severely dense opacity obscuring the details of the intraocular structures.

^b^The severity of bullous keratopathy was defined as follows: stage 1, slight stromal edema or increased corneal thickness due to reduction in corneal endothelial cells; stage 2, stromal edema with DM folds; stage 3, stromal edema and DM folds with apparent epithelial bullae; stage 4, stromal scarring and corneal neovascularisation.

### Corneal topographic map patterns

**Figure 2:**
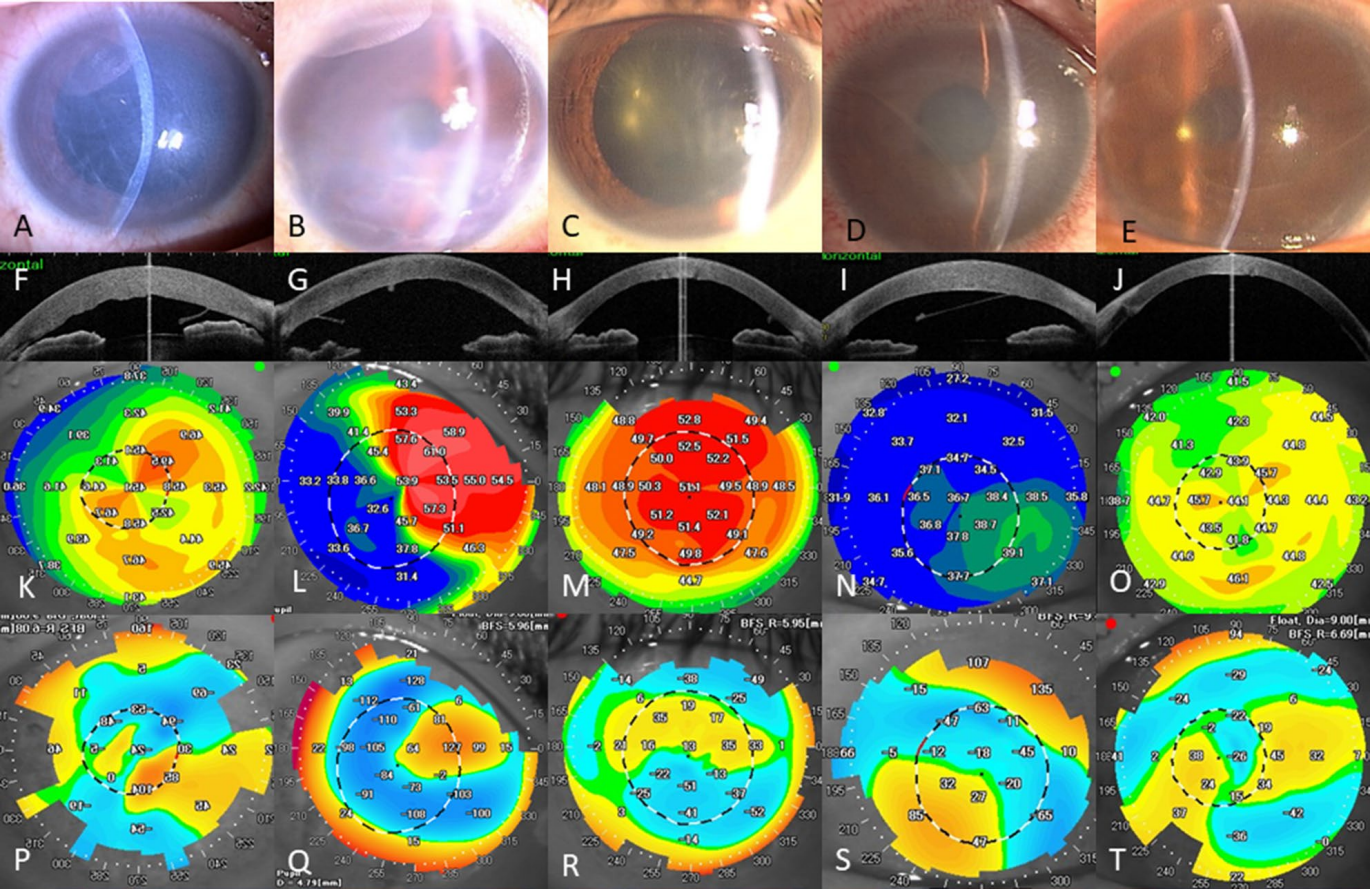
Representative cases of forceps injuries. Representative slit-lamp photographs (**A**–**E**), AS-OCT images (**F**–**J**), and topographic maps of the anterior (**K**–**O**) and posterior surfaces (**P**–**T**) of eyes with bullous keratopathy due to forceps corneal injuries. Topographic maps are characterized into five patterns: regular astigmatism (**A**, **F**, **K** and **P**), asymmetry (**B**, **G**, **L**, and **Q**), protrusion (**C**, **H**, **M**, and **R**), flat (**D**, **I**, **N**, and **S**), and posterior irregular patterns (**E**, **J**, **O**, and **T**).

**Figure 3:**
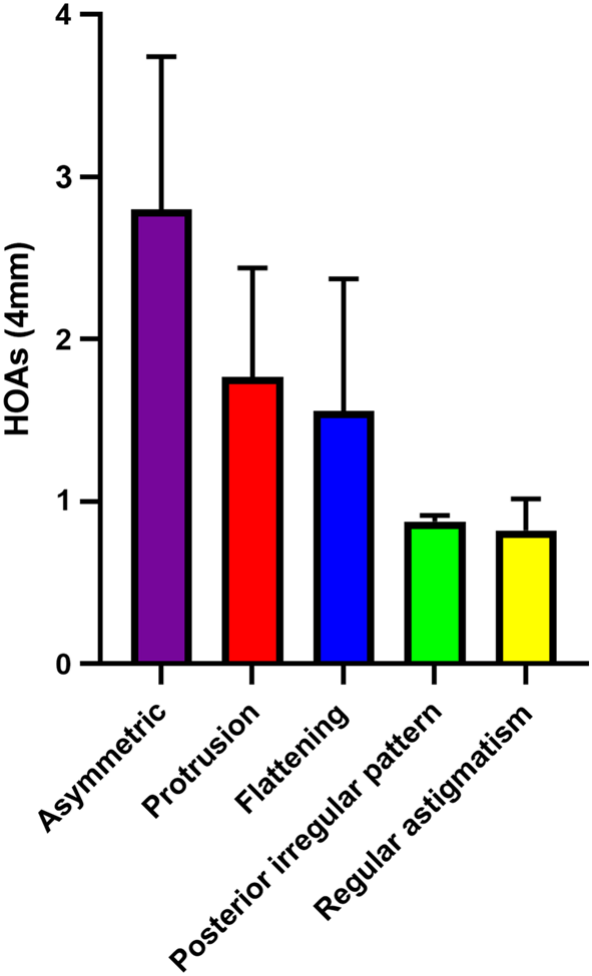
Comparison of corneal HOAs among various topographic map patterns. HOAs were measured for asymmetric, protrusion, flattening, regular astigmatism, and posterior irregular pattern respectively. HOAs tended to be higher for asymmetric, protrusion, and flattening than for regular astigmatism and posterior irregular pattern. However, due to small number, there was no significant statistical difference in each topographic map pattern.

Representative cases are shown in Fig. 2. Protrusion and regular astigmatism patterns were identified as the most common alterations in the topographic maps (both, 6 eyes, 26.1%), followed by asymmetric pattern (5 eyes, 21.7%), flattening pattern (4 eyes, 17.4%), and posterior irregular pattern with minimal anterior surface changes (2 eyes, 8.7%). As shown in Fig. 3, HOAs stratified by corneal shape were higher for asymmetry, protrusion, and flattening patterns than those for posterior irregular and regular patterns, although significant differences were not observed due to a small number of subjects in each pattern.

### Corneal higher-order aberrations before and after corneal transplantation

To evaluate the influence of corneal oedema in eyes with forceps corneal injury, we assessed corneal HOAs before and after DSAEK. After DSAEK, logMAR visual acuity was improved from 1.52 (0.61–1.70) (median [IQR]) to 0.52 (0.40–0.76) (*P* < 0.01, Table 4). Corneal HOAs were significantly improved after DSAEK (Table 4). Although there were no statistically significant correlations presumably due to the small number of subjects, logMAR visual acuity after DSAEK was weakly correlated with corneal HOA (6 mm, *r*_*s*_ = 0.59, *P* = 0.06). Among the 23 eyes, three patients (four eyes) underwent penetrating keratoplasty (PKP). LogMAR visual acuity was significantly improved from 1.90 ± 0.17 to 0.60 ± 0.24 after PKP (P < 0.01). HOAs of the posterior corneal surface were significantly decreased from 0.83 ± 0.06 to 0.57 ± 0.69 after PKP (P < 0.01, Supplementary Table).

**Table 4:** Corneal higher-order aberrations before and after DSAEK in eyes with bullous keratopathy due to forceps corneal injuries.

	Before DSAEK	After DSAEK	*P* value*
BSCVA (logMAR)	1.52 (0.61, 1.70)	0.52 (0.40, 0.76)	** < 0.01**^α^
HOA 4 mm			
Total	1.01 (0.93, 1.32)	0.79 (0.61, 0.87)	**0.02**^α^
Anterior	0.91 (0.84, 1.09)	0.62 (0.58, 0.81)	**0.02**^α^
Posterior	0.49 (0.45, 0.60)	0.31 (0.23, 0.43)	0.08^α^
HOA (6 mm)			
Total	1.63 (1.48, 2.09)	1.43 (1.19, 1.96)	0.70^α^
Anterior	2.00 (1.62, 2.19)	1.60 (1.30, 2.03)	0.20^α^
Posterior	0.77 (0.59, 0.85)	0.70 (0.66, 0.99)	0.32^α^
SA (4 mm)			
Total	0.72 (0.50, 0.92)	0.49 (0.32, 0.52)	**0.04**^α^
Anterior	0.61 (0.58, 0.81)	0.41 (0.27, 0.42)	** < 0.01**^α^
Posterior	0.39 (0.22, 0.45)	0.22 (0.17, 0.28)	0.16^α^
SA (6 mm)			
Total	1.35 (1.27, 1.53)	0.72 (0.64, 0.90)	0.55^α^
Anterior	0.98 (0.80, 1.24)	0.66 (0.56, 0.78)	**0.01**^α^
Posterior	0.39 (0.35, 0.51)	0.44 (0.39, 0.52)	0.35^α^
Coma (4 mm)			
Total	0.77 (0.61, 0.91)	0.63 (0.47, 0.69)	**0.04**^α^
Anterior	0.70 (0.54, 0.90)	0.56 (0.46, 0.68)	0.11^α^
Posterior	0.36 (0.28, 0.47)	0.22 (0.16, 0.30)	0.10^α^
Coma (6 mm)			
Total	1.35 (1.27, 1.53)	1.24 (0.99, 1.74)	0.77^α^
Anterior	1.72 (1.35, 1.92)	1.49 (1.09, 1.92)	0.47^α^
Posterior	0.63 (0.40, 0.73)	0.55 (0.47, 0.60)	0.30^α^

Data is shown by median (IQR) μm in 11 patients who underwent DSAEK.

BSCVA, best-spectacle corrected visual acuity; DSAEK, Descemet stripping automated endothelial keratoplasty; IQR, interquartile range; logMAR, logarithm of minimal angle resolution; SA, spherical aberration.

^α^*P* values: Mann–Whitney test. Bold numbers indicate *P* < 0.05.

## Discussion

Forceps corneal injury causes vision loss later in life and pain due to epithelial bulla formation associated with bullous keratopathy. As it is a rare disease, accounting for only 1–2% of bullous keratopathy cases^4^, the clinical subtypes and degrees of astigmatism in eyes with forceps corneal injury remains poorly understood. The current study showed increased corneal HOAs, SA, and coma aberrations in eyes with bullous keratopathy due to forceps corneal injury. This study also showed that coma aberrations were associated with decreased visual acuity.

To our knowledge, this was the largest case series to have quantified corneal aberrations and clinical data in eyes with forceps corneal injuries. Owing to the small number of cases, previous studies have included samples of only one to 12 patients^1–3,6–8,19–26^. Therefore, it has been difficult to accurately characterise clinical features such as the degree of regular and irregular astigmatism, incidence of amblyopia, corneal structure, and optical properties. For example, since Lloyd reported six forceps corneal injuries in which the left eye was the affected eye in all cases^27^, it was believed that the left eye is often traumatised during the process of passing through the birth canal^27^; however, in this study, the injured eye was more often the right (57.1%) than the left (42.9%). Forceps corneal injury causes amblyopia because of a high degree of corneal astigmatism^7^. In this case series, 22 of 23 patient eyes had amblyopia. Furthermore, we found that the direction of DM breaks coincided with the steep meridian of the cornea in 14 of 23 eyes (60.9%) with apparent astigmatism axis with regular and flattening topographic patterns, whereas in eyes with asymmetric and protrusion patterns, there was no association with the astigmatism axis. Interestingly, the topographic elevation map of the anterior and posterior surface showed that the corneal posterior shift was located exactly in the area of the DM breaks (Fig. 1C, G, K) suggesting that DM breaks directly cause astigmatism in the posterior surface.

**Figure 4:**
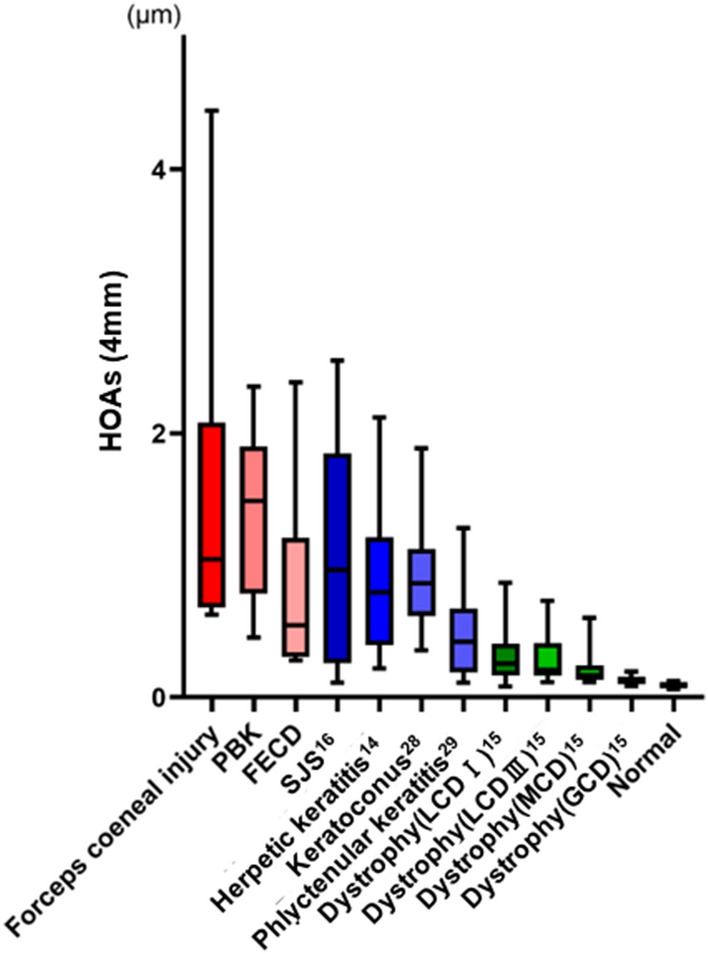
Comparison of corneal HOAs among various corneal diseases. In the previous studies^14–16,28,29^, the amount of HOAs in eyes with forceps corneal injury was larger than that in eyes with other corneal diseases. PBK, pseudo phakic bullous keratopathy; FECD, Fuchs’ endothelial corneal dystrophy; SJS, Stevens Johnson syndrome; LCDI, lattice corneal dystrophy type I; LCDIII, lattice corneal dystrophy type III; MCD, macular corneal dystrophy; GCD, granular corneal dystrophy.

When comparing the HOAs of the total cornea (4 mm) based on our previous studies on various corneal diseases, including pseudophakic bullous keratopathy and Fuchs’ endothelial corneal dystrophy (Fig. 4)^14–16,28,29^, the corneal HOAs differed among various corneal diseases with large intra-individual differences (large standard deviation). Notably, previous studies showed that visual acuity was significantly correlated with corneal HOAs in all corneal diseases, except granular corneal dystrophy type 2, where HOAs were not increased^15^. In forceps corneal injuries, the mean HOA was much higher than that in other diseases. Moreover, it is noteworthy that the corneal HOAs of the posterior surface were significantly larger in eyes with forceps corneal injury. Theoretically, in healthy eyes, where the posterior and anterior surfaces are parallel to each other, aberrations of the posterior surface tend to compensate for those of the anterior surface, resulting in fewer corneal HOAs in the total cornea^30^. The disruption of parallelism between the anterior and posterior surfaces in forceps corneal injuries may cause the lack of compensation, leading to increased total corneal HOAs. Measurement of corneal HOAs is clinically relevant because it is correlated with visual acuity. We believe that corneal HOAs can comprise a universal diagnostic biomarker for decision-making in corneal transplantation, and can be applied to various corneal diseases, including forceps corneal injury.

In the current study, we found that both the posterior surface, where the DM scroll is located and the anterior surface had large aberrations in eyes with forceps corneal injury (Fig. 2, Table 2). When considering surgical indications, DASEK is a standard surgical treatment for forceps corneal injury because of its several advantages over PKP, including rapid visual recovery, biomechanical properties, and integrity^31–33^. However, in DSAEK, large amounts of HOAs on the anterior surface can remain after surgery and adversely affect visual acuity. In the current case series, after corneal transplantation (PKP in four eyes of three patients, and DSAEK in 11 patients), although there were significant improvements in HOAs and logMAR visual acuity (Table 4 and Supplementary Table), the improvement in visual acuity was limited especially in patients with large HOAs due to stromal deformation from forceps corneal injury. Thus, we need to evaluate corneal HOAs, when deciding whether to perform PKP or DSAEK in eyes with bullous keratopathy after forceps corneal injury, especially when considering early intervention in eyes with mild corneal oedema.

This study had several limitations. First, the patient age at onset of bullous keratopathy due to forceps corneal injury could have caused bias. Age has been reported to affect visual function, refraction, and astigmatism^34^. However, age-related HOA changes are minor, compared with the increase in corneal HOAs in eyes with forceps corneal injury. Therefore, we believe that the age-related bias was minimal. Second, there might be some differences in the refractive indices between normal corneas and bullous keratopathy, which may have influenced our estimated HOAs. Theoretically, corneal oedema can affect refractive index and errors in refractive power of the cornea by less than 5%^35,36^, minimally influencing the results of the current study. Third, 22 eyes (95.7%) in current study had amblyopia. It should be emphasized that severity of amblyopia may have influenced the analysis and is an important factor limiting postoperative visual acuity. However, a correlation between visual acuity and corneal aberrations was observed despite the presence of amblyopia.

In conclusion, Descemet’s scrolls in eyes with forceps corneal injuries induce morphometric changes in the anterior and posterior surfaces, leading to increased corneal HOAs, SA, and coma of the total cornea and anterior and posterior surfaces. We also classified irregular astigmatism patterns into five types, based on the corneal topographic map. The most common patterns are protrusion and regular astigmatism. Furthermore, increases in coma were significantly correlated with visual acuity, indicating that coma may be a biomarker for assessing visual function in eyes with forceps corneal injuries.

## Supplementary Information

Supplementary Information.

## Data Availability

The datasets used and/or analysed during the current study available from the corresponding author on reasonable request. Meeting presentation: We presented this study in ARVO2022.
