# Supplementary material for: Corneal higher-order aberrations in corneal endothelial decompensation secondary to obstetric forceps injury
Source: Sci Rep. 2023 Apr 3;13:5389. doi: 10.1038/s41598-023-32683-5 (PMC10070416; doi:10.1038/s41598-023-32683-5)
Supplement: Supplementary file 1 — Supplementary Information. [file 41598_2023_32683_MOESM1_ESM.pdf]

## Methods

*Penetrating keratoplasty*

We performed penetrating keratoplasty (PKP) in four eyes according to our standard techniques, as previously published.[1] Briefly, PKP was performed under retrobulbar anesthesia. The donor button was cut with a Barron punch trephine (diameter, 7.75 mm in all eyes). The recipient bed was 7.5 mm in all cases. A Hessburg–Barron suction trephine was used to cut a partial-depth, circular incision in the cornea, centered at the geometric center of the cornea. Excision of the recipient corneal button was completed with curved corneal scissors. The graft was sutured in place with a single-running 10-0 nylon suture with 24 bites.

## Results

*Corneal higher-order aberrations before and after PKP*

LogMAR visual acuity was significantly improved from 1.90 ± 0.17 to 0.60 ± 0.24 after PKP ($P < 0.01$). HOAs of the posterior corneal surface were significantly decreased from 0.83 ± 0.06 to 0.57 ± 0.69 after PKP ($P < 0.01$, Supplementary Table).

## Reference

1. Yamaguchi T, Ohnuma K, Tomida D, et al. The contribution of the posterior surface to the corneal aberrations in eyes after keratoplasty. Invest Ophthalmol Vis Sci 2011;52(9):6222-9.

**Supplementally table 1.** Corneal higher-order aberrations before and after PKP in eyes with bullous keratopathy due to forceps corneal injuries

| | Before PKP (3 eyes) | After PKP (3 eyes) | *P* value[*] |
|---|---|---|---|
| **BSCVA (logMAR)** | 1.90 ± 0.17 | 0.60 ± 0.24 | **<0.01**[α] |
| **HOAs (4mm)** | | | |
| Total | 3.42 ± 1.31 | 3.52 ± 0.61 | 0.91[α] |
| Anterior | 3.52 ± 1.64 | 3.83 ± 0.58 | 0.77[α] |
| Posterior | 0.83± 0.06 | 0.57 ± 0.69 | **<0.01**[α] |
| **HOAs (6mm)** | | | |
| Total | 5.83 ± 0.90 | 7.64 ± 0.58 | 0.17[α] |
| Anterior | 6.30 ± 2.49 | 8.97 ± 1.00 | 0.20[α] |
| Posterior | 1.27 ± 0.57 | 2.12 ± 1.37 | 0.38[α] |
| **SA (4mm)** | | | |
| Total | 2.01 ± 1.73 | 1.28 ± 0.29 | 0.51[α] |
| Anterior | 2.13 ± 2.02 | 1.48 ± 0.37 | 0.61[α] |
| Posterior | 0.55 ± 0.18 | 0.34 ± 0.02 | 0.11[α] |
| **SA (6mm)** | | | |
| Total | 3.14 ± 1.92 | 4.02 ± 0.65 | 0.49[α] |
| Anterior | 3.39 ± 2.21 | 4.50 ± 0.94 | 0.47[α] |
| Posterior | 0.73 ± 0.22 | 1.19 ± 1.01 | 0.49[α] |
| **Coma (4mm)** | | | |
| Total | 2.54 ± 0.75 | 3.28 ± 0.55 | 0.24[α] |
| Anterior | 2.51 ± 0.97 | 3.53 ± 0.48 | 0.18[α] |
| Posterior | 0.59 ± 0.13 | 0.45 ± 0.09 | 0.19[α] |
| **Coma (6mm)** | | | |
| Total | 4.62 ± 1.73 | 6.45 ± 1.23 | 0.21[α] |
| Anterior | 4.98 ± 2.51 | 7.71 ± 1.80 | 0.20[α] |
| Posterior | 1.02 ± 0.57 | 1.74 ± 0.98 | 0.33[α] |

Data is shown by mean ± SD µm

BSCVA: best-spectacle corrected visual acuity, HOAs: higher-order aberrations, PKP: penetrating keratoplasty, logMAR: logarithm of minimal angle resolution, SA: spherical aberration, SD: standard deviation.

[α]*P* values: Mann–Whitney test.

Bold numbers indicate $P < 0.05$.
